# 

**Published:** 1843-08

**Authors:** 


### CONTENTS

### OF NO. II.

---

### ORIGINAL COMMUNICATIONS.

### ESSAYS AND CASES.

Art. I.—Case of Cancer.—Reported to the Medical Society of Tennessee, May, 1843. By A. H. Buchanan, M.D., of Nashville, Tennessee. - - - - 81
Art. II.—Cases in Surgery.—Reported to the Medical Society of Tennessee. By John M. Esselman, M.D., of Nashville, Tennessee. - - - 88
Art. III.—Singular effects of Ipecacuanha.—A Case reported to the Medical Society of Tennessee. By Felix Robertson, M.D., of Nashville, Tennessee. - 95
Art. IV.—An Account of an Osseous Deposit in the Dura Mater. Read before the Medical Society of Tennessee. By J. W. Stout, M.D., of Nashville, Tennessee. - - - - - - - 97
Art. V.—A Case of Intermittent from Birth, and its subsequent progress.—Reported to the Medical Society of Tennessee. By Dr. B. W. Avent, of Murfreesborough, Tennessee. - - - - - 101
Art. VI.—A Case of Functional, Simulating Organic Disease of the Heart. By H. R. Roberts, M.D., Columbia, Tennessee. - - - - - 104

Art. VII.—Some Account of the Disease called by the people "Black Tongue," which prevailed in some parts of Missouri, in the winter of 1842–'3. By Dr. E. H. Bennett, of New Madrid, Missouri. - - 110

REVIEWS.

Art. VIII.—Tenth Annual Report of the State Lunatic Hospital, at Worcester, Massachusetts, December, 1842. Boston, 1843: 8vo., p. 115, &c., &c., &c. - 114

SELECTIONS FROM AMERICAN AND FOREIGN JOURNALS.

Gun-shot Wound. Extensive laceration of the Brain, without loss of consciousness, or impairment of mind. - - 126
Electro-Magnetism a Remedy for Opium Poisoning. - 129
Fracture of the Pelvis—Laceration of the Bladder—Recovery. 130
Universal Suppuration of the Cerebro-spinal Membranes, without any corresponding symptoms. - - - 130
Typhus Abdominalis. - - - - - 132
Case of Strangulated Intestine, from Rotation of the Sigmoid Flexure—with remarks. - - - - 133
Medical Writers Good Practitioners. - - - 139
Structure of Neuromatous Tumours in Stumps. - - 141
Treatment of Pneumonia. - - - - 142
Extract of a Lecture on Syphilis. - - - - 143
A Curiosity in Obstetric Physiology. - - - 147
Structure of the Uterus. - - - - - 149
Ferruginated Pill of Mercury. - - - - 150
Indications afforded by the state of the Iris in cases of Cerebral Lesion. - - - - - - 151

ORIGINAL INTELLIGENCE.

Travelling Editorials. - - - - - - 153
The Influenza. - - - - - - 159
Death of Judge Rowan. - - - - - - 159
Gross on Wounds of the Intestines and Artificial Anus. - 160

